# Time to death and its determinants of under-five children in rural Ethiopia by using shared frailty

**DOI:** 10.1038/s41598-024-56063-9

**Published:** 2024-03-07

**Authors:** Getahun Dejene Yemane, Abiyu Abadi Tareke, Hamdi Fekredin Zakaria, Bayley Adane Takele, Sebwedin Surur Jemal

**Affiliations:** 1 Department of Statistics, College of Natural and Computational Science, Mizan-Tepi University, Tepi, Ethiopia; 2 Zonal COVID-19/EPI Technical Assistant at West Gondar Zone Health Department, Amref Health Africa in Ethiopia, Gondar, Ethiopia; 3 Department of Epidemiology and Biostatistics, School of Public Health, Haramaya University, Harar, Ethiopia; 4 Department of Epidemiology and Biostatistics, Institute of Public Health, College of Medicine and Health Science, University of Gondar, Gondar, Ethiopia

**Keywords:** Survival status, Under-five mortality, Shared frailty, Health care, Signs and symptoms

## Abstract

Under-five (U5M) is one of the most significant and sensitive measures of the community's health. Children who live in rural areas are more likely than those who live in urban areas to die before the age of five. Therefore, the study aimed to assess the Survival status of under-five mortality and its determinants in rural Ethiopia. The 2019 Ethiopia Mini Demographic and Health Survey was used in this study as a secondary source (EMDHS). A total of 4426 weighted under-five children were included in the study. To determine survival time and identify predictors of death among children under the age of five, the Cox's gamma shared frailty model and the Kaplan Meier model, respectively, were used. An adjusted Hazard Ratio (AHR) along with a 95% Confidence Interval (CI) were used to measure the size and direction of the association. The Study showed that in rural Ethiopia, 6.03% of children died before celebrating their first birthday. The median age of under-five mortality in rural Ethiopia was estimated to be 29 Months. The hazard of death among under-five children and those who had given birth to two children in the last five years was 4.99 times less likely to be at risk of dying than those who had given birth to one Child in the previous five years (AHR 4.99, 95% CI 2.97, 8.83). The Study Concluded that under-five mortality remained high in rural Ethiopia. In the final model, the Age of Mothers, Sex of Household, Breastfeeding, Types of Birth, Sex of Child, Educational Level of Mothers, Wealth Index, Child ever born, Marital Status, and Water Source were significant predictors of under-five mortality. Twins and children who are not breastfed should receive additional attention, along with improving water resources for households and mothers income.

## Introduction

Under-five Mortality (U5M) is the likelihood that a child born in a certain year or period will pass away before becoming five years old, according to the prevailing age-specific mortality rates during that time. For countries and organizations around the world, the high death rate among children has remained a major worry. Worldwide, there were almost 5.3 million baby and child fatalities in 2018. Eight times greater than in the WHO European zone, the risk of under-five mortality in the WHO Africa region was 76 deaths per 1000 live births^1^.

Sub-Saharan Africa continues to have the highest mortality rate for children under five worldwide. More than 80% of the 5.2 million deaths of children under the age of five that were reported worldwide in 2019 occurred in SSA and Central and Southern Asia. Nigeria, India, Pakistan, the Democratic Republic of the Congo, and Ethiopia together account for half of all under-five mortality in 2019^2,3^. By 2030, the under-five mortality rate must be reduced to at least 25 per 1000 live births, according to Sustainable Development Goal (SDG) 3 objective 3.2 of the United Nations (UN)^4,5^. The majority of SSA nations are not on track to reach this goal for under-five mortality objects of SDG-3^6^. Senegal, Tanzania, Uganda, Kenya, Rwanda, and Senegal are the five nations on track to achieve the SDG target of reducing the under-5 mortality^6^. According to a study conducted in Ethiopia, children who live in rural areas are more likely than those who live in urban areas to die before they reach the age of five^7,8^. According to a survival analysis of under-five mortality in Nigeria, the place of residency had a substantial impact on the probability of under-five death^9^. Numerous studies have shown that living in a rural location is more frequently linked to a higher risk of under-five mortality but the rural disadvantage in U5M is still there, as the last review demonstrated. Therefore, policies and programs targeted at eliminating rural disadvantage need to be assessed. The rural setting has continuously been linked to higher U5M^11,12^. This may be a result of subpar child care practices, restricted access to competent medical care, inadequate transportation, a lack of community health knowledge, and delays faced by rural households seeking medical care.

At least 80% of Ethiopians lived in rural areas, and the average age and life expectancy were both quite low for those in rural areas^13,14^. Ethiopia set a goal to lower the U5MR to 25 or less per 1000 live births by 2030^15^. This objective won't be met if the risk factors are not carefully identified and dealt with. Rural Ethiopia was not the focus of an earlier study. All of the studies on under-five mortality conducted in Ethiopia focused on both urban and rural populations, despite the discovery of significant differences^16,17^. Furthermore, the data showed a correlation even though it had been ignored in past studies on the subject^16,18,19^. The analysis did not take into account the influence of time on under-five mortality since it is time to the critical event, even if some research made an effort to account for the nature of the data^20,21^. The ideal way to describe these types of data is to take both filtering and hierarchical data into consideration^10,22–24^. In order to account for the linked nature of the data in rural areas, the study aimed to assess the Time to death and its determinants in rural Ethiopia by using Shared frailty analysis.

## Study population and data source

The survey was conducted from March 21, 2019, to June 28, 2019, by the Ethiopian Public Health Institute (EPHI), working in collaboration with the Central Statistical Agency (CSA), the Federal Ministry of Health (FMOH), and under the general direction of the Technical Working Group (TWG). Following formal registration, the DHS program made the 2019 Ethiopian Mean Demographic Health Survey (EMDHS) data available. The study population consisted of all children under-five years old who were selected from certain clusters in rural Ethiopia. The data collection, which was limited to children under five living in rural regions, recorded both the surviving and deceased people's ages at the time of death.

### Study design

Cross-sectional community-based study Design.

### Dependent variable

Time to under-five deaths in months.

### Independent variable

Maternal reproductive and obstetric characteristics (mothers age at first birth, place of delivery, Types of birth, and birth order), socioeconomic and demographic factors (mother education, wealth index of the household, and availability of water sources), and infant characteristics made up the independent variables (sex of the child).

### Inclusion and exclusion criteria

Included all Ethiopian children under the age of five who lived in rural areas but eliminated urban dwellers and those with inadequate information about the incident or its timing.

### Data analysis

Data analysis was carried out using the Stata/SE version 14.0. The statistical significance of variations between categorical predictor variables in the result of interest across time was examined using the Log-rank test. The time to mortality for children under five was also calculated using the Kaplan–Meier method. The significant contribution of clusters (frailty) on time to child mortality and the overall model significance tests were evaluated using the Likelihood ratio chi-square and Wald chi-square tests, respectively. The Cox-gamma shared frailty model was used to find predictors of death in children under the age of five. Given that the data were grouped at the cluster level, shared gamma frailty analysis was performed. The theta chi-square test was used to quantify between-group heterogeneity, and the results were significant (P < 0.001). So, this between-group heterogeneity creates unexploded variations.

The analysis started with a single-variable cox-gamma regression model, and variables for multivariable cox-regression were chosen if their p-value was less than 0.2. Following that, the analysis was carried out in four steps: Null Model, which had no explanatory variables; Model I, which contained only individual-level variables; Model II, which contained just clusters; Model III, which contained community-level variables; and Model IV (both individual and community-level variables). AHR (Adjusted Hazard Ratio) and random effect, as well as their 95% Confidence Interval (CI), were used to assess the measure of association (fixed effect). The level of statistical significance in the final model was set at P < 0.05. Cox-Snell residuals and a Nelson Aalen cumulative hazard function versus the Cox-Snell residuals plot were used to assess the model. Also, the log-likelihood ratio deviance test was used.

### Ethical consideration and consent statement

The Ethiopian Health and Nutrition Research Center (EHNRI) Review Board, the National Research Ethics Review Committee (NRERC) at the Ministry of Science and Technology, the Institutional Review Board of Inner-City Fund (ICF) International, and the Centers for Disease Control and Prevention provided ethics clearance and consent to participate in the 2019 EMDHS (CDC). The Ethiopian Health and Nutrition Research Center (EHNRI) Review Board waived the need for obtaining informed consent, however the information was kept private and anonymous. The Helsinki Declaration carried out this investigation.

## Results

### Sociodemographic characteristics of the respondents

The total number of under-five children included in the analysis was 4426. In rural Ethiopia, the estimated median time for under-five mortality was 29 months. From the total of under-five Children, 2298 (51.9%) were male and 2128 (48.1%) were female. Regarding breastfeeding status, 1320 (29.8%) children were not breastfeeding at all. 248 (5.6%), eighty hundred Sixty-one (19.5%), and 936 (21.1%) of the children were born to Mothers aged between 15–19, 20–24, and 30–34 respectively. Similarly, more than half (61.2%) of under-five children had to breastfeed. Regarding Birth Type, 4293 (97%) of the mothers were born with one child during their pregnancy. 774 (17.5%) of the households were headed by women. 2299 (51.9%) of the children were male. The majority of the respondents 1688(38.1%) had no shared Toilet service facility, and 511 (11.5%) had shared toilet service (Table 1).

**Table 1:** Household and parental characteristics of under-five children in rural Ethiopia based on the 2019 EMDHS.

Variable	Frequency	Percentage	Chi-square log rank test	P-value
Age in 5 years group				
15–19	248	5.6	2.94	0.08
20–24	861	19.5		
25–29	1411	31.9		
30–34	936	21.1		
35–39	600	13.6		
40–44	277	6.3		
45–49	93	2.1		
Sex of household head				
Male	3652	82.5	1.67	0.19
Female	774	17.5		
Births in the last five years				
1	1733	39.2	44.46	0.00
2	2100	47.4		
3	546	12.3		
4	32	0.7		
5	15	0.3		
Currently breastfeeding				
No	1320	29.8	30.42	0.00
Yes	2708	61.2		
Child is twin				
Single birth	4293	97.0	82.10	0.00
1st of multiple	65	1.5		
2nd of multiple	65	1.5		
3rd of multiple	3	0.1		
Sex of child				
Male	2298	51.9	2.64	0.10
Female	2128	48.1		
Toilet facilities shared with other households				
No	1688	38.1	1.52	0.22
Yes	511	11.5		
Not resident	36	0.8		
Region				
Tigray	381	8.6	6.55	0.01
Afar	546	12.3		
Amhara	456	10.3		
Oromia	632	14.3		
Somali	527	11.9		
Benishangul	461	10.4		
SNNPR	601	13.6		
Gambela	356	8.0		
Harari	243	5.5		
Dire Dawa	223	5.0		
Addis Ababa				

### Time to under-five mortality (Kaplan–Meier estimates of failure function)

**Figure 1:**
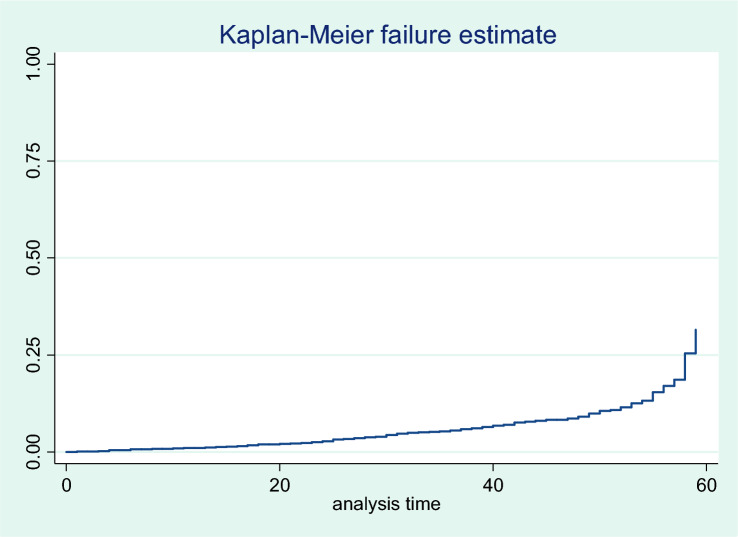
Kaplan–Meier estimates of failure function of under-five mortality in rural Ethiopia.

The findings showed that 5.73% and 5.54% of children died before 28 days and before celebrating their first birthday, respectively, in rural Ethiopia. Similar to this, the Kaplan–Meier estimate's findings revealed that 6.03% of children in rural Ethiopia died before turning five, as shown in the picture below. The rate of mortality among children under five was 3.2 per 100 person-months, while the average and median time to death were, respectively, 29.94 and 30 months for all deaths. (Fig. 1).

### Factors of under‑five mortality in rural Ethiopia

The Cox-gamma shared frailty model indicated that Age of Mothers, Sex of Household, Breastfeeding, Types of Birth, Sex of Child, Educational Level of Mothers, Wealth Index, Child ever born, Marital status and Water Source were significant Predictors Independent of under-five mortality.

The hazard of death among under-five children and those who had birthed two children in the last five years was 4.99 times less likely to be at risk of dying than those who had birthed one Child in the last five years (AHR 4.99, 95% CI 2.97, 8.83). The hazard of death among under-five children who had breastfed for a long period was decreased by 57% as compared to their counterparts (AHR 5.69, 95% CI 2.28, 14.22). Under-five children who had twin births were 12.42 times more likely to be at risk of death as compared to singletons (AHR 12.42, 95% CI 6.13,25.16). The hazard of death among under-five male children was increased by 12.5% as compared to female children (AHR 12.5, 95% CI 1.15, 8.21). The hazard of death among under-five children of primarily educated mothers was decreased by 40% as compared to uneducated Mothers (AHR 0.40, 95% CI 0.25, 0.66). Under-five children who had twin births were 12.42 times more likely at risk of death as compared to singleton (AHR 12.42, 95% CI 6.13, 25.16). The hazard of death among under-five children and those who were born in Richer households decreased by 40% as compared to those who were born in the poorest households (AHR 0.40, 95% CI 0.20, 0.80). The hazard of death among under-five children and their mothers had were married decreased by 34% as compared to Women never in union (AHR 0.34, 95% CI 0.22, 0.45). The hazard of death among under-five children and those who had Shared toilet service was 2.28 times more likely to be at risk of dying than those who had no Shared Toilet Service (AHR 2.28, 95% CI 1.43, 12.19). The hazard of death among under-five children and those who had unimproved water sources was 3.84 times more likely at risk of dying than those who had improved Water Sources (AHR 3.84% CI 1.13, 13.04). The risk of under-five mortality was 5.95 times higher among children whose mothers had born more than five children (AHR 5.95, 95% CI 3.42, 10.34) (Table 2).

**Table 2:** Shared frailty survival analysis factors affecting under-five death using the recent 2019 EMDHS data. (n = 4426).

Variable	Null model	Model I CHR 95% CI	Model II AHR (95% CI)	Model III AHR (95% CI)	Model IV AHR (95% CI)
Age					
15–19		1			1
20–24		5.40 (1.18, 24.67)	1.06 (0.55, 2.08)		0.72 (0.57, 0.92)
25–29		3.49 (0.84, 14.42)	0.48 (0.26, 0.86)		0.69 (0.55, 0.87)
30–34		3.59 (0.88, 14.58)	0.27(0.15, 0.48)		0.66 (0.52, 0.84)
35–39		3.00 (0.73, 12.32)	0.32 (0.176, 0.584)		0.60 (0.47, 0.77)
40–44		2.95 (0.71, 12.26)	0.32(0.14, 0.52)		0.66 (0.49, 0.89)
45–49		1.78 (0.39, 8.03)	0.31 (0.14, 0.66)		0.46 (0.31, 0.69)
Sex of HH					
Male		1			1
Female		1.03 (0.75, 1.43)	1.24 (0.86, 1.79)		0.70 (0.50, 0.99)
Current Breastfeeding					
No		1			1
Yes		1.32 (0.99, 1.75)	4.88 (3.58, 6.65)		5.69 (2.28, 14.22)
Types of birth					
Single		1			1
Two		7.91 (5.22, 11.97)	0.48 (0.12, 1.97)		12.42 (6.13, 25.16)
Three		25.53 (8.14, 80.07)	1.10 (0.29, 4.12)		1.70 (1.13, 2.57)
Sex of child					
Male		1			1
Female		1.32 (0.49, 3.58)	1.32 (1.02, 1.70)		1.25 (1.15, 8.21)
Education					
No education		1			1
Primary		1.17 (0.78, 1.73)	1.42 (1.08, 1.86)		0.40 (0.25, 0.66)
Secondary		1.57 (1.15, 2.14)	0.85 (0.42, 1.72)		0.25 (0.16, 0.41)
Higher		2.14 (0.92, 4.99)	0.73 (0.23, 2.36)		0.24 (0.15, 0.40)
Wealth index					
Poorest					
Poorer		0.89 (0.65, 1.22)	0.85 (0.62, 1.17)		0.51 (0.30, 0.87)
Middle		0.90 (0.64, 1.27)	0.84 (0.59, 1.19)		0.32 (0.18, 0.59)
Rich		0.81 (0.55, 1.18)	0.75 (0.51, 1.11)		0.40 (0.20, 0.80)
Richest		0.70 (0.38, 1.30)	0.66 (0.35, 1.24)		0.42 (1.20, 0.92)
Children ever born					
0–2		1			1
3–5		1.12 (0.82)	0.75 (0.55, 1.03)		2.63 (1.27, 5.40)
> 6		0.83 (0.62, 1.10)	0.93 (0.67, 1.29)		5.95 (3.42, 10.34)
Marital status					
Never union		1			1
Married		3.30 (0.46, 23.57)			0.34 (0.22, 0.45)
Living with partner		3.36 (0.30, 37.03)			0.79 (0.20, 0.83)
Separated		2.70 (0.28, 25.93)			20.23 (3.24, 22.35)
Divorced		5.59 (0.73, 42.77)			3.5 (2.25, 3.75)
Region					
Tigray		1			1
Affar		2.47 (1.18, 5.20)		2.57 (0.61, 10.78)	1.40 (1.27, 7.38)
Amhara		1.97 (0.91, 4.29)		1.59 (0.44, 5.80)	3.42 (2.79, 14.86)
Oromia		2.65 (1.28, 5.47)		2.60 (0.78, 8.58)	1.26 (1.24, 4.94)
Somalia		3.49 (1.68, 7.26)		1.67 (0.41, 6.76)	6.40 (3.42, 28.85)
Benishangul Gumuz		3.85 (1.87, 7.90)		3.52 (1.08, 11.5)	1.59 (1.32, 7.85)
SNNPR		1.88 (0.88, 3.10)		1.62 (0.48, 5.47)	2.19 (1.43, 11.14)
Gambella		3.326 (1.58, 7.00)		3.86 (1.14, 13.1)	0.83 (0.39, 1.76)
Harar		2.75 (1.19, 6.35)		1.64 (0.42, 6.35)	0.50 (0.23, 1.05)
Addis Ababa		3.33 (1.48, 7.46)		2.32 (0.60, 8.97)	0.45 (0.20, 1.01)
Source of water					
Improved				1	1
Unimproved				0.82 (0.64, 1.04)	3.84 (1.13, 13.04)

## Model fitness and adequacy test

**Figure 2:**
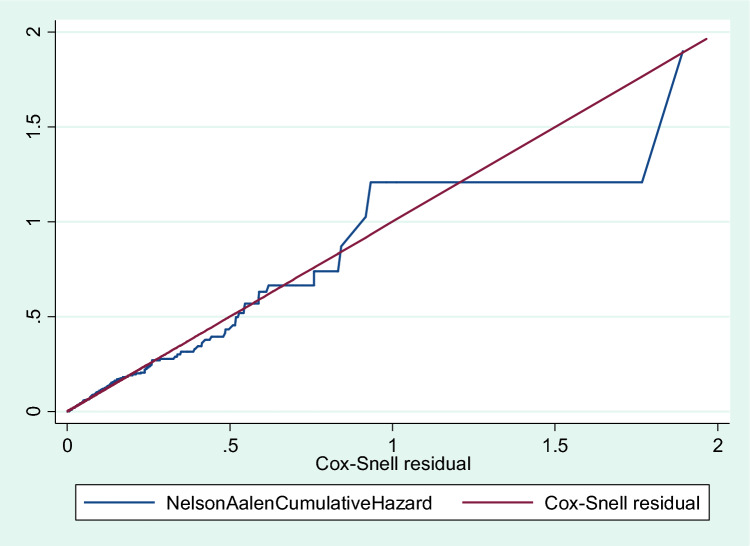
Cox-Snell residuals plot for fitting Cox-model for both individual and community-level Characteristics of Under-five mortality in rural Ethiopia.

The final model was the one that fit the data with the most accuracy compared to the other models because it had the smallest log-likelihood (Table 3) and (Fig. 2).

**Table 3:** Model fitness and adequacy test statistics for all level predictors of under-five mortality in rural Ethiopia.

Model fitness and adequacy checking	Null model	Model I	Model II	Model II
Log-likelihood	− 1693.89	− 1666.08	− 870.67	− 735.76
LR test for device	1	0.0000	0.0000	0.0000

## Discussion

This Finding showed that 5.73%, 5.54%, and 6.03% of children died before 28 days, before celebrating their first and fifth birthdays, respectively, in rural Ethiopia. The finding was higher than a study conducted in Ghana (4.91%)^25^ and Bhutan (3.70%)^26^. This finding is lower than the study conducted in rural Ethiopia using the 2016 Ethiopian demographic and health survey^23^ and the study done in Chad (13%)^27,28^. The finding was also lower than a study conducted in Tanzania (7.04%)^29^. The socioeconomic position of the nation, the availability of universal healthcare, the study's timing, and the location of the study are all potential sources of variation. This Study showed that child who had not breastfed had higher risk of under-five mortality than those who had breastfed for a long time. Longer breastfeeding periods have been demonstrated to lower the incidence of under-five mortality in previous research^30–32^. Breastfeeding provides the newborn with enough natural nutrients, shields them from various illnesses, and strengthens their immune systems^33^. Previous investigations have also validated this finding^34–36^. This may be due to the fact that breastfeeding can lower morbidity and help avoid under-five infection-related deaths^37^. Since early introduction to exclusively breastfeeding functioned as the starting point for continuum care for the mother by encouraging bonding, giving colostrum as the baby's first vaccine, and infants that can have a long-lasting impact on health and brain development for decades.

This Study was showed that Male Children had a higher risk of under-five mortality than female Children. The results were consistent with research done in Ethiopia^19,22,38–40^. The outcome was comparable to research done in Malawi^41^, Ghana^25^, and Nigeria^42^. Additionally, a study done in Sierra Leone^43^ and Tanzania^29^. Male child mortality is higher than female child mortality, which can be attributed to biological factors (such as lower resistance to infection, increased risk of preterm birth, and difficult labor due to a larger average body size and head circumference), gender discrimination in feeding and medical care practices, or a reaction to HI-related drugs^44,45^.

This finding showed that a child born to mothers with no education had a higher risk of under-five mortality than their counterparts. This study was in agreement with studies on mortality in a children under five^20,42,46–51^. Compared to their counterparts, mothers who have received education appear to be more rational and conscious of the value of using healthcare, eating well, and maintaining good hygiene. Furthermore, it is noted that lower maternal and father education are both is a significant factor in under-five mortality even after controlling the family's socioeconomic situation^52,53^.

Another protective factor essential to the child's survival is the mother's age at delivery. Young moms' biological and social mechanisms have a negative impact on their first child's health. A child born to adolescent moms has precarious health outcomes and has a greater chance of dying before age five^54^. Different studies^55,56^ compared to women who had children between the ages of 20 and 34, have shown that mothers who were younger (typically under 20 years old) when they gave birth had significantly higher odds of having a child who died before age five; however, there is no specific age group to which these findings should be categorized.

This Finding Showed that Family size is significant factors of under-five Mortality in Rural Ethiopia. This conclusion was accepted after previous research found that, compared to households with 1–2 children, those with 3–5 and 6 children saw greater rates of child mortality^57^. When compared to children born as singletons, children born as multiple births had a lower chance of surviving. Mothers may pay their kids less attention, and it's likely that they don't receive enough nutrition or adequate medical treatment. As a result, in order to lower child mortality, governments should focus more of their attention on preventing grand parity.

This Finding showed that the likelihood of under-5 mortality was higher among single mothers compared with married mothers. Similar results have been attained in investigations carried out in SSA^57,58^ and other developing nations^26,59^. Most studies have found that the high prevalence of under-5 mortality among children born to single parents is mostly caused by a lack of marital support^45,57,60^. Other studies have linked inadequate nutrition, which can result in stunting, wasting, and underweight and endanger children's lives, to the increased risk of under-5 mortality among children born to single moms as opposed to married mothers^61,62^.

This result demonstrated that families led by women played a lower risk of under-five mortality than households headed by men. Adhikari and Podhisita reported similar findings, indicating that households headed by women had a lower incidence of under-five mortality^63^. This demonstrates how women's empowerment and autonomy through increased maternal literacy and their capacity to make autonomous decisions on the use of maternity healthcare services, including pediatric care, could contribute to a decrease in under-five mortality.

This result demonstrated that children from wealthy and middle-class homes had a lower risk of under-five mortality compared to those from poorer households. Similar research in 55 low-resource nations indicated that households with lower economic status had considerably higher rates of under-five child mortality than those with greater economic status. These studies used the household assets index to quantify wealth status^10,24,58,64^. Access to goods and services like food, shelter, transportation, or financial access to care may have had a role in how household wealth status affected under-five children's mortality^65^.

This result demonstrated the regional variation in under-five mortality. Numerous studies have discovered a strong correlation between geographic location and under-five mortality^30,55,66^. Such variations in under-five mortality across the region may be brought about by unequal access to healthcare facilities or by varying levels of interventions, policies, and programs for childhood survival. In order to close the gap, it is essential to investigate the causes and create intervention measures. Numerous studies have demonstrated the negative correlation between a family's economic situation and under-five mortality. Compared to wealthy families, poor families are constrained to have greater rates of under-five mortality^45,51^. This discrepancy could exist for a number of reasons. Poorly off families struggle to pay for expensive medical care when it is necessary, may not give mothers enough nutritious food, may not know how to care for their child's and mother's overall health, etc.

This result demonstrated that those who used improved water had a decreased risk of under-five mortality than people who used unimproved water. This conclusion was supported by research done in Nigeria^67^, Egypt, and Eritrea^68,69^. This finding could be explained by the fact that as kids begin to crawl and walk, they come into contact with viruses that cause diarrhea from a range of environmental sources, such as tainted water^70^. Additionally, households frequently use unimproved water to make weaning foods, which exposes kids to microorganisms that cause diarrheal illness, which has a high fatality rate^71,72^.

## Limitations

Self-reported interviews that were conducted at a different time point were used to generate cross-sectional data, which is subject to recall bias and social desirability bias. It was anticipated that the disadvantage of secondary data would exist.

## Conclusion

Newborns had a very low chance of surviving because the risk of death for children under the age of five rose rather than fell with age. Age of Mothers, Sex of Household, Breastfeeding, Types of Birth, Sex of Child, Educational Level of Mothers, Wealth Index, Child ever born, Marital Status, and Water Source were significant predictors of mortality in children under five. Given the foregoing, we propose that health care programs be created particularly to promote a healthy family structure. To improve maternity and child health services and increase access to potable water in the area, government and non-governmental groups must collaborate. To improve the child's health and the health of the family as a whole, healthcare professionals should get involved in the community.

## Data Availability

On www.measuredhs.com, you can access the datasets developed and/or examined for the current investigation. But if you register legally at www.measuredhs.com and send a persuasive letter to the project for the DHS program, you can view the study's data. The corresponding author can be contacted for a reasonable fee for the datasets used and/or analyzed during the current work.
